# Peritoneal and hematogenous metastases of ovarian cancer cells are both controlled by the p90RSK through a self-reinforcing cell autonomous mechanism

**DOI:** 10.18632/oncotarget.6412

**Published:** 2015-11-26

**Authors:** Erica Torchiaro, Annalisa Lorenzato, Martina Olivero, Donatella Valdembri, Paolo Armando Gagliardi, Marta Gai, Jessica Erriquez, Guido Serini, Maria Flavia Di Renzo

**Affiliations:** ^1^ Department of Oncology, University of Torino School of Medicine, Turin, Italy; ^2^ Candiolo Cancer Institute, Fondazione del Piemonte per l'Oncologia (FPO)–Istituto di Ricovero e Cura a Carattere Scientifico (IRCCS), Candiolo, Italy; ^3^ Department of Molecular Biotechnologies and Health Sciences, University of Turin at the Molecular Biotechnology Center, Torino, Italy

**Keywords:** p90RSK, peritoneal metastasis, hematogenous metastasis, cell adhesion, fibronectin

## Abstract

The molecular mechanisms orchestrating peritoneal and hematogenous metastases of ovarian cancer cells are assumed to be distinct. We studied the p90RSK family of serine/threonine kinases that lie downstream the RAS-ERK/MAPK pathway and modulate a variety of cellular processes including cell proliferation, survival, motility and invasiveness. We found the RSK1 and RSK2 isoforms expressed in a number of human ovarian cancer cell lines, where they played redundant roles in sustaining *in vitro* motility and invasiveness. *In vivo*, silencing of both RSK1 and RSK2 almost abrogated short-term and long-term metastatic engraftment of ovarian cancer cells in the peritoneum. In addition, RSK1/RSK2 silenced cells failed to colonize the lungs after intravenous injection and to form hematogenous metastasis from subcutaneous xenografts. RSK1/RSK2 suppression resulted in lessened ovarian cancer cell spreading on endogenous fibronectin (FN). Mechanistically, RSK1/RSK2 knockdown diminished FN transcription, α5β1 integrin activation and TGF-β1 translation. Reduced endogenous FN deposition and TGF-β1 secretion depended on the lack of activating phosphorylation of the transcription/translation factor YB-1 by p90RSK. Altogether data show how p90RSK activates a self-reinforcing cell autonomous pro-adhesive circuit necessary for metastatic seeding of ovarian cancer cells. Thus, p90RSK inhibitors might hinder both the hematogenous and the peritoneal metastatic spread of human ovarian cancer.

## INTRODUCTION

Ovarian cancer is the most lethal gynecological cancer. Because early disease is asymptomatic, it is diagnosed at advanced stage, when cancer cells have invaded tissues beyond the ovaries [1]. Clinical retrospective studies suggest that epithelial ovarian carcinomas spread very efficiently within the peritoneal cavity, but more rarely metastasize outside of it. It has been generally thought that, once ovarian cancer cells have detached as single cells or clusters from the primary ovarian tumor, they metastasize, likely through a passive mechanism, to the peritoneum and particularly to the omentum. Thus, this biological behavior of ovarian carcinoma seemed to be unique, differing markedly from classic well-studied pattern of hematogenous metastasis found in most other cancers, where cancer cells go through several steps of intravasation and extravasation before establishing metastases within other organs. However, it has been shown that the formation of peritoneal nodules by ovarian cancer cells may also be a natural consequence of their hematogenous dissemination [2]. Moreover, the in-depth analysis of the biology of ovarian cancer cells showed how their preferential omental implantation is specific and sustained by cytokines produced by the omentum [2, 3].

Several protein kinases have been implicated in mediating prometastatic signaling in human cancers and attention is focused on them because they are the most amenable targets for therapy. *In vitro* and *in vivo* evidence suggests a role of the 90 kDa ribosomal S6 kinases (RSKs) in the metastatic process [for a review see 4]. RSK1 has been identified as a common downstream effector for multiple distinct stimuli activating cell migration [5] and able to promote invasion of nodular melanoma cells [6]. Overexpression of constitutively active RSK2 has been shown to elicit a motile phenotype in MDCK cells [7]. *In vivo*, RSK2 knockdown decreased the ability of head and neck squamous cell carcinoma to metastasize to lymph nodes [8, 9].

RSKs are a family of Ser/Thr kinases that lie downstream the RAS/MAPK cascade [for reviews see 10, 11]. This family consists of four human isoforms encoded by four different genes that share high degree of sequence homology and are all characterized by the presence of two distinct kinase domains sequentially activating one another, both of which are catalytically functional. RSKs are activated by the extracellular signal regulated kinases (ERK1/2) in response to many growth factors, hormones and neurotransmitters. Indeed, by acting both at transcriptional and post-transcriptional levels, RSK proteins play also roles in the control of cell cycle and survival.

The four RSK1–4 kinase isoforms are expressed at different extent in several cell lines as well as normal and cancer tissues [11, 12]. RSK1 and RSK2 are the best characterized RSK family members. Indeed, overexpression of RSK1 and RSK2 kinases has been reported in a number of human cancers, including breast and prostate tumors [13, 14]. RSK1 and RSK2 are likely activated in ovarian cancer, because of the frequent activation of receptors such as HER2, MET, EGFR and LPARs [15]. In this context, by means of functional *in vitro* and *in vivo* assays, we studied the role of RSK kinases in the growth of metastatic nodules of ovarian cancer cells in either the peritoneal cavity or in distant organs. We show here that the RSK1 and RSK2 kinases play a key role in the homing of ovarian cancer cells in metastatic sites by regulating cell adhesion and invasion likely through a mechanism involving the RSK1/2-driven activation of the transcription/translation factor YB-1, the transcription of the FN1 gene and the translation of the TGF-β1 mRNA.

## RESULTS

### RSK isoforms in ovarian cancer cell lines

**Figure 1:**
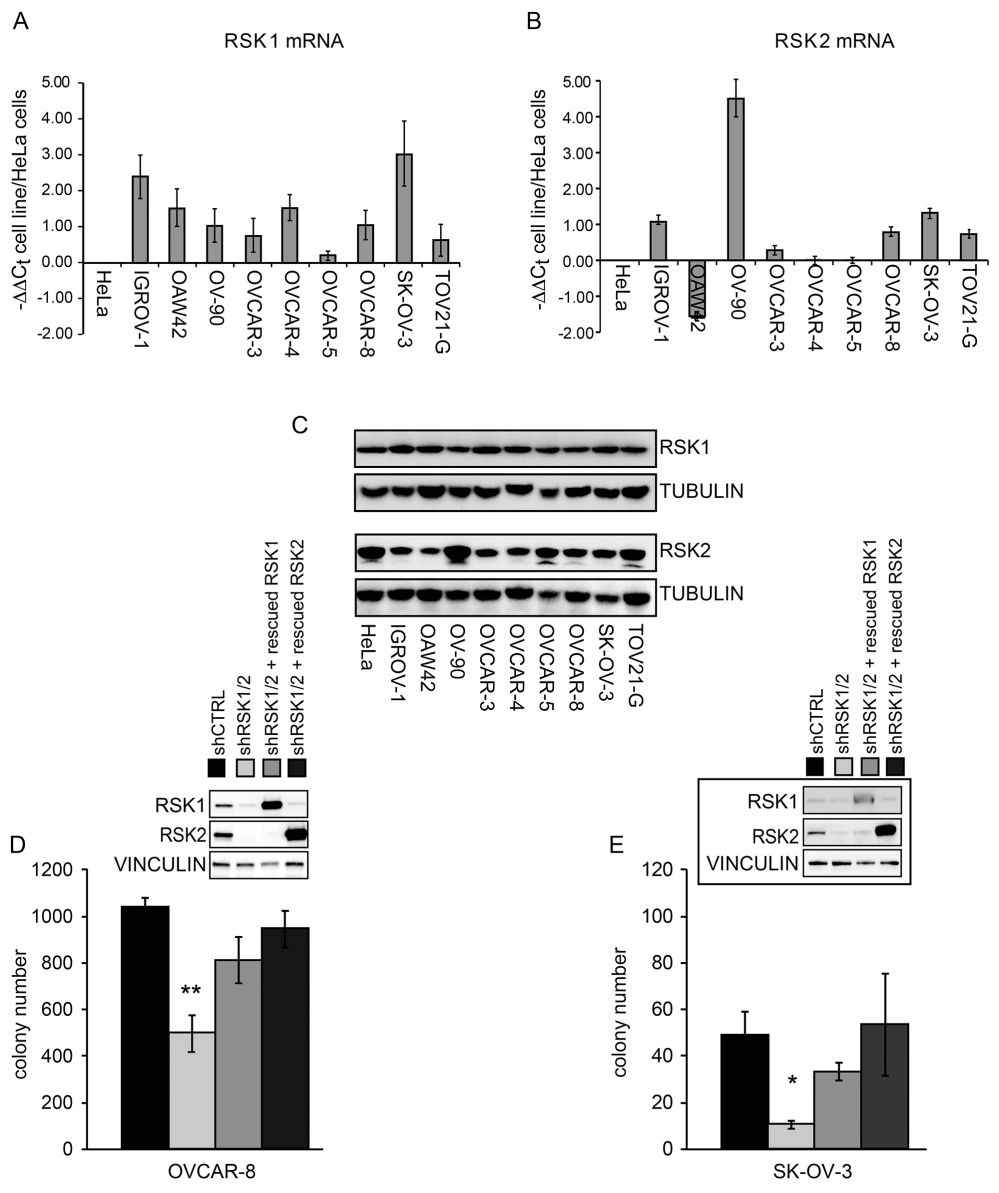
RSK1 and RSK2 are expressed in ovarian cancer cells and play role in anchorage independent growth *in vitro* **A.** Quantitative Real-time PCR (qPCR) of RSK1 expression in the listed ovarian cancer cell lines, using HeLa cells, derived from a cervical adenocarcinoma, as reference cell line; **B.** qPCR of RSK2 expression in the listed ovarian cancer cell lines, using HeLa as reference cell line; **C.** Western blot analyses showing RSK1 and RSK2 protein expression; blots were reprobed with tubulin antibody to confirm equal loading; **D–E.** growth in semisolid agar medium of OVCAR-8 and SK-OV-3 cells where both RSK1 and RSK2 were silenced with each form specific shRNAs (shRSK1/2). As control, cells were transduced with a scramble shRNA (shCRTL). To assess the contribution of each isoform, either RSK1 or RSK2 function was rescued in RSK1/RSK2 silenced cells by the stable expression, driven by lentiviral vector, of either human cDNA carrying eight silent mutations that rendered the corresponding mRNA unaffected by interference. Statistical significance was determined using ANOVA test: *P < 0.05, **P < 0.01.

Each of the four RSK isoforms is not equally expressed in all cell types [11, 12]. We evaluated their expression in nine ovarian cancer cell lines at both mRNA and protein level. As shown in Figure 1A–1C, in most cell lines RSK1 and RSK2 are expressed at a level comparable to that of a reference cell line, such as the HeLa cell line. Conversely RSK3 and RSK4 were expressed at very low level or almost undetectable in the same ovarian cancer cell lines (Supplementary Figure S1), as in most of the ovarian cancer cell lines analyzed and reported in the Cancer Cell Line Encyclopaedia (CCLE) [16] (Supplementary Figure S2).

It is known that the RSKs are involved in the control of proliferation of several cell lines through direct effect on cell cycle proteins and through indirect effects, e.g. through transcription factor activation [11, 12]. To assess the functional role of RSK1 and RSK2 in proliferation, in the SK-OV-3 and OVCAR-8 cell lines the two genes were silenced in combination by means of specific shRNAs carried by lentiviral vectors. The combined RSK1 and RSK2 silencing allowed to rule out the compensatory effect due to the other isoform when only one of them is silenced. The compensatory expression of RSK3 and RSK4 was also ruled out (Supplementary Figure S3). Moreover, to evaluate the specificity of the silencing and to appreciate the individual contribution of each isoform, we separately re-expressed RSK1 and RSK2 in doubly RSK1/RSK2 silenced cells. The rescue was obtained by the stable lentiviral vector-driven expression of either human cDNA carrying eight silent mutations that rendered the corresponding mRNA unaffected by interference (Figure 1D–1E, insets). Supplementary Figure S4 shows that the proliferation of the doubly silenced cells as well as those where each isoform was rescued was modestly affected by the silencing. Conversely, the combined RSK1/RSK2 silencing reduced the ability of the same cell lines to grow in semisolid agar medium (Figure 1D–1E), suggesting a prevailing role of RSK1 and RSK2 in anchorage independent growth of ovarian cancer cells. Individual rescue experiments showed that RSK1 and RSK2 kinases played redundant roles in enabling cell anchorage-independent growth (Figure 1D–1E).

### Suppression of both RSK1 and RSK2 affects motility and invasiveness of ovarian cancer cells *in vitro*

**Figure 2:**
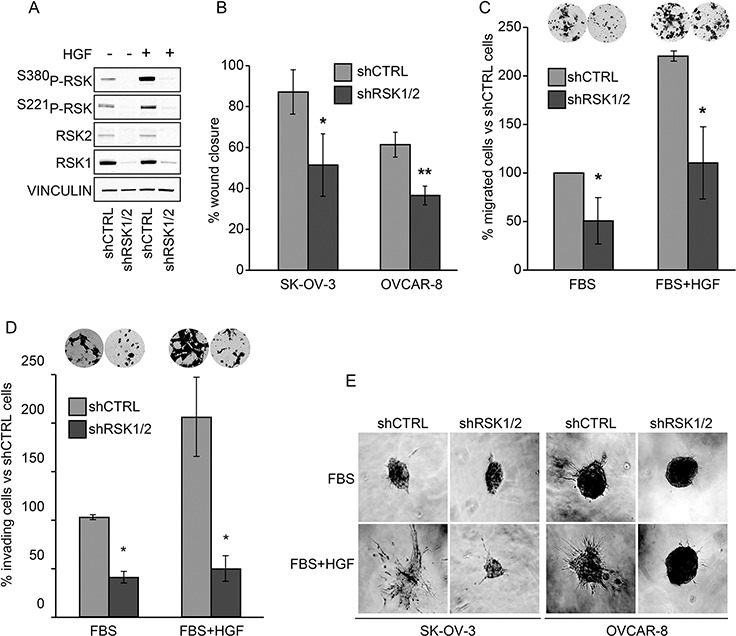
RSK1 and RSK2 double knockdown impairs motility and invasiveness of ovarian cancer cells *in vitro* Both RSK1 and RSK2 (shRSK1/2) were silenced with specific shRNAs in SK-OV-3 and OVCAR-8 cells. Control cells were obtained by transduction with non-targeting short hairpin sequence (shCTRL). **A.** Western blot analysis of the basal and HGF stimulated (50 ng/ml) activation of RSK1 and RSK2; phospho-specific antibodies recognized both the RSK1 and RSK2 phosphorylated at the two critical serine residues; blots were re-probed with vinculin antibody to confirm equal loading. **B.** Wound healing assay in the presence of foetal bovine serum (FBS) plus HGF (50 ng/ml); 24 hours after wounding, cells that migrated to the acellular area were photographed under the microscope and percentage of closure was measured with ImageJ software. **C.** Migration through Transwell^®^ permeable supports of control or doubly silenced OVCAR-8 towards FBS or towards FBS plus HGF (50 ng/ml). Migrated cells (insets) were photographed after 16 hours and counted with ImageJ software. **D.** Matrigel^®^ invasion assay: migration through Matrigel^®^-coated filters of control and doubly silenced OVCAR-8 cells in the presence of FBS or FBS plus HGF (50 ng/ml). The lower side of the filters (insets) was photographed after 16 hours and cells were quantified using the ImageJ software. **E.** Three-D collagen invasion assay: cells were left to aggregate in methylcellulose. The obtained spheroids were embedded in 3D collagen gel containing FBS or FBS plus HGF as above and photographed after 24 hours. Statistical significance was determined using ANOVA test: *P < 0.05. **P < 0.01.

Both RSK1 and RSK2 regulate epithelial cell motility and invasiveness [5, 7]. As many growth factors activate p90RSK in cancer, we evaluated whether RSK1/RSK2 silencing might impair the motile phenotype of ovarian cancer cells induced by Hepatocyte Growth Factor (HGF) that is known also as Scatter Factor, for its ability to trigger cell motility and invasiveness and ultimately to stimulate cell metastatic ability [17, 18]. As expected, HGF activated p90RSK (Figure 2A). Figure 2B–2C show that the RSK1 and RSK2 double knockdown reduced the HGF triggered motility of ovarian cancer cells in functional *in vitro* assays, such as wound closure and directional migration. To assess the specificity of silencing and the individual contribution of RSK1 and RSK2, the expression of each isoform was rescued as above. Supplementary Figure S5A shows that the rescue of either RSK1 or RSK2 alone was sufficient to fully revert the inhibition of motility due to RSK1/RSK2 silencing.

RSK1/RSK2 double-knockdown also impaired the ability of ovarian cancer cells to invade an artificially reconstituted basement membrane made of collagen, laminin, and glycosaminoglycans (Matrigel^®^) covering Transwell pores (Figure 2D). Moreover, combined RSK1/RSK2 silencing almost abrogated the ability of ovarian cancer cells to invade a three dimensional collagen gel (Figure 2E). This assay highlights the potential of cells to invade another type of surrogate extracellular matrix. The individual rescue of either RSK1 or RSK2 in doubly silenced cells showed that the kinases play redundant roles in cell invasiveness as well (Supplementary Figure S5B–S5C–S5D).

### RSK1 and RSK2 silencing impairs ovarian cancer cell ability to grow as peritoneal nodules *in vivo*

The role of RSK1 and RSK2 in cell motility and invasion suggested that these kinases might be involved in the metastatic spread of ovarian cancer cells. The latter preferentially metastasize to intra-abdominal organs, mostly because cells detach from the primary site, which can be the Fallopian tube or the ovary, and disseminate to the adjacent peritoneum. Thus, the ability of ovarian cancer cells to grow as peritoneal nodules was assayed in short-term [19] and long-term [20] colonization assays, which had been shown to be indicative of the capability of these cells to adhere/invade and to grow into the peritoneum, respectively.

As RSK1 and RSK2 played fully redundant roles in cell motility and invasiveness assays *in vitro*, both kinases were silenced in OVCAR-8 cells by transducing cells to express either both shRNAs for RSK1 and RSK2 or a control non-targeting short hairpin sequence (shCTRL). To allow fluorescence monitoring of metastasis, shRNAs were delivered using a lentiviral vector also carrying the TurboGFP transgene.

**Figure 3:**
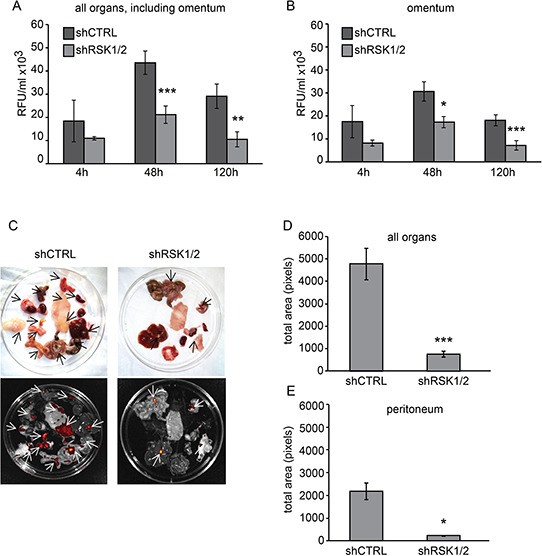
RSK1 and RSK2 silencing impairs the ability of ovarian cancer cells to form peritoneal metastases *in vivo* To assay the short-term and long-term ability of ovarian cancer cells to form metastases in the peritoneum, 4 × 10^6^ OVCAR-8 control (shCTRL) or RSK1/RSK2 (shRSK1/2) silenced cells, all transduced using lentiviral vectors also carrying the TurboGFP transgene, were injected intra-peritoneum in NOD/SCID mice. **A–B.** For the short-term assay, after 4, 48 and 120 hours (h) mice were sacrificed and omentum, peritoneum, diaphragm and abdominal fat were lysed with 1% NP40. Fluorescence intensity was quantified (RFU: relative fluorescence units) using a spectrophotometer (Synergy HT) in lysates of (A) all organs including the omentum and of the omentum (B). For long-term colonization assay the mice were sacrificed after 21 days **C-D-E.** Panel (C) shows representative images of metastases to intra-abdominal organs visualized (arrows) and quantified (D-E) by analyzing fluorescent emission using IVIS Lumina II imaging system and the ImageJ software, in all organs (D) and in the peritoneum alone (E): diaphragm, peritoneum, intestine, fat, stomach, spleen, liver, omentum and kidney. Statistical significance was determined using ANOVA test: *P < 0.05, **P < 0.01, ***P < 0.001.

For the short-term assay, cells were injected intra-peritoneum (i.p.) and after 4, 48 and 120 hours the amount of fluorescent cells attached to the peritoneum was measured (Figure 3A–3B). As early as 4 hours after the injection the number of doubly silenced cells that remained attached to the peritoneum was lower than that of control cells, but the difference was statistically significant only after 48 and 120 hours. Metastases of both control and RSK1/RSK2 silenced cells were mostly localized in the omentum (Figure 3B).

Long-term assay, based on the sacrifice and analyses of animals 21 days after the i.p. injection of cells, showed that peritoneal homing and growth of metastatic nodules was strongly reduced by RSK1/RSK2 silencing (Figure 3C–3D–3E).

Altogether these data show that the growth of ovarian cancer cells in the peritoneum as metastatic nodules was affected by RSK1/RSK2 silencing.

### RSK1 and RSK2 silencing impairs the ability of ovarian cancer cells to form hematogenous metastases *in vivo*

**Figure 4:**
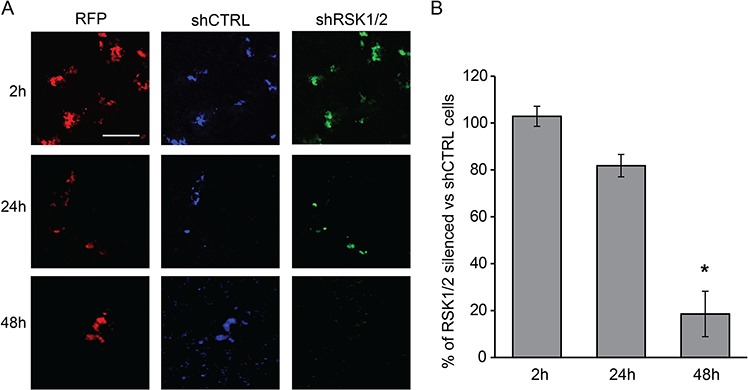
RSK1 and RSK2 silencing impairs the ability of ovarian cancer cells to form haematogenous metastases *in vivo* Cell ability to form lung colonies was measured with a short-term assays based on the i.v. injection of RSK1/RSK2 silenced and control cells and monitoring of cell localization after 2, 24 and 48 hours. Before i.v. injection, control cells and RSK1/RSK2 silenced cells, both carrying also the TurboRFP (Red Fluorescent Protein, RFP) transgene as a marker of infection, were differentially labelled with cellTraceTM VIOLET (violet shCTRL cells) and CFSE (green shRSK1/2 cells) reagents, respectively. Equal numbers of cells were then mixed and injected intravenously; after 2, 24 and 48 hours (h) lungs were explanted and fixed. Cells were visualized with two-photon confocal microscopy. **A.** representative fields of the lungs. **B.** graph representing ratio between green and violet fluorescent cells in the lungs. Lungs of three mice for each time point were examined and at least four fields per lung were acquired. For each field a Z-stack acquisition of several slices was made. For image analysis with ImageJ software, a maximum intensity projection was made for all fields before quantifying the area covered by cells for each single channel. Statistical significance was determined using ANOVA test: *P < 0.05. Scale bar: 100 μm.

To assay the intrinsic ability of ovarian cancer cells to colonize the lung, we used a short term lung colonization assay. Control and RSK1/RSK2 silenced cells were labeled with different fluorescent vital dyes, co-injected in equal number into the tail vein of immunocompromised mice and then monitored for their ability to colonize the host lungs. Figure 4 shows that after 2 and 24 hours control and RSK1/RSK2 silenced cells were present in the lungs at a ratio 1:1. This reflects the mechanical entrapment of cells in the lung rather than their local adhesion [21]. Conversely, 48 hours after injection RSK1/RSK2 silenced cells were less than 20% of total cells localized in the lungs (Figure 4). At this time point trapped cells are partially dislodged and only those able to adhere and invade remain localized in the lung. This explains the persistence of a small number (20%) of still mechanically trapped silenced cells.

We then assayed the role played by RSK1 and RSK2 in the *in vivo* control of spontaneous metastatic dissemination of cells growing as subcutaneous xenografts. As *in vitro* motility and invasiveness was strongly activated by HGF, a circulating growth factor that is considered a poor prognostic marker in ovarian cancer patients [22], control and silenced cells were further engineered to secrete HGF in order to enhance their metastatic potential. Supplementary Figure S6A documents the effectiveness of RSK1/RSK2 silencing and of HGF expression.

Although we found that RSK1/RSK2 silenced tumors grew almost comparably *in vitro* (Supplementary Figure S4), to definitively rule out a possible effect of the double knockdown on tumor growth, shRNA expression was obtained with an inducible vector and induced four weeks after the subcutaneous injection of engineered cells (Supplementary Figure S6A–S6B).

Four weeks after the induction of RNA interference, local muscle wall invasion and spontaneous lung metastases were observed in 7/7 mice with control subcutaneous xenografts and in only 1/7 mice with RSK1/RSK2 silenced xenografts (Supplementary Figure S6C–S6D). The efficacy of RSK1 and RSK2 silencing in xenografts was confirmed, as shown in Supplementary Figure S6E–S6F.

Altogether these data showed that RSK1/RSK2 silencing almost suppressed the ability of ovarian cancer cells to form experimental hematogenous metastases.

### In ovarian cancer cells RSK1 and RSK2 silencing impairs a pro-adhesive circuit made of fibronectin, α5β1 integrin and TGF-β1

*In vivo* hematogenous and peritoneal metastasis assays suggested that RSK1/RSK2 kinases are required for ovarian cancer cell adhesion to vessel walls and peritoneal surfaces.

**Figure 5:**
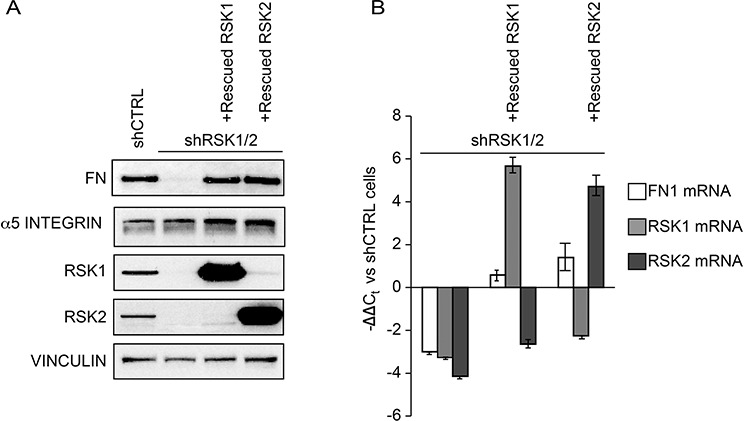
RSK1 and RSK2 silencing affects FN but not integrin α5 chain expression **A.** Western blot analysis of integrin α5 chain and fibronectin (FN) in cells silenced and rescued as described in the Legend to Figure 1; blots were re-probed with vinculin antibody to confirm equal loading. **B.** Quantitative Real-time PCR of the expression of the listed genes in cells silenced and rescued as in the Legend to Figure 1.

**Figure 6:**
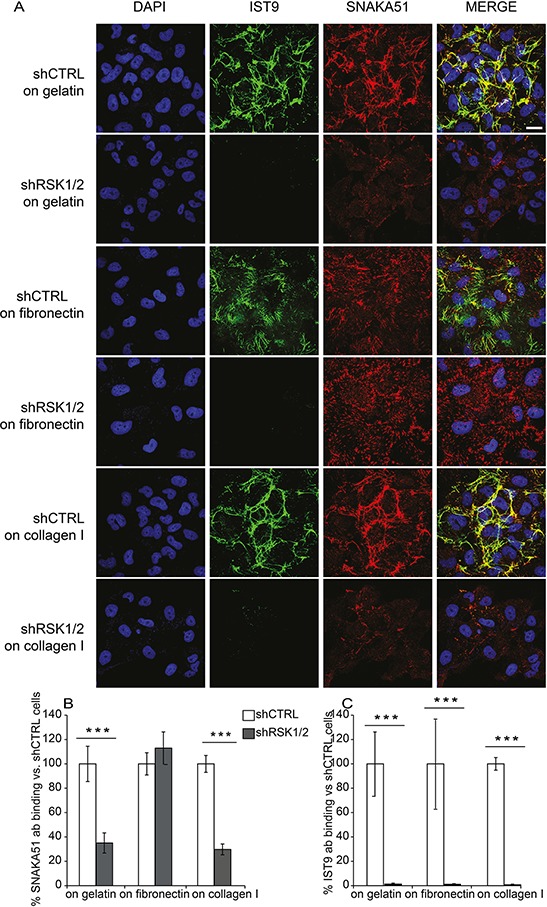
RSK1 and RSK2 silencing affects adhesive proteins in ovarian cancer cells **A.** Immunofluorescence confocal analysis of FN deposition and α5β1 integrin activation in control and RSK1/RSK2 silenced cells; DAPI was used to visualize cell nuclei. FN was labelled with IST9 monoclonal antibody specific for cell derived FN; active α5β1 integrin was revealed incubating living cells with SNAKA51 monoclonal antibody; **B.** SNAKA51 and **C.** IST9 antibody (ab) binding measurement obtained by quantifying pixel intensity in cellular region of interest. Average pixel intensity was normalized to pixel intensity of shCTRL cells. Standard deviation represents the variability of average pixel intensity in different fields. Scale bar: 20 μm.

In ovarian cancer [19, 23], as in many physiological and pathological conditions, α5β1 integrin-mediated cell adhesion to fibronectin (FN) plays an important role in controlling cell motility and promoting metastasis [see e.g. ref. 24]. We hence evaluated the expression of endogenous FN and α5β1 integrin in ovarian cancer cells. Endogenous cellular FN, as recognized by the IST-9 mouse monoclonal antibody (mAb) [25], was expressed and secreted by control ovarian cancer cells (Figure 5A–5B and 6A). Notably, cellular *FN1* gene transcription (Figure 5B) as well as FN protein synthesis (Figure 5A), secretion and polymerization (Figure 6A–6C) were instead almost abrogated by RSK1/RSK2 silencing and fully rescued by either kinase re-expression (Figure 5A–5B). In addition, while α5 integrin subunit protein expression levels were not affected by RSK1/RSK2 silencing (Figure 5A), the activation of the major FN receptor α5β1 integrin, monitored by means of the conformation-specific anti-α5 integrin subunit mouse mAb SNAKA-51 [26, 27], was reduced as well (Figure 6A–6B). Activation of α5 integrin was FN dependent, as it was restored by plating cells on exogenous FN, but not on collagen I (Figure 6A–6B–6C)

**Figure 7:**
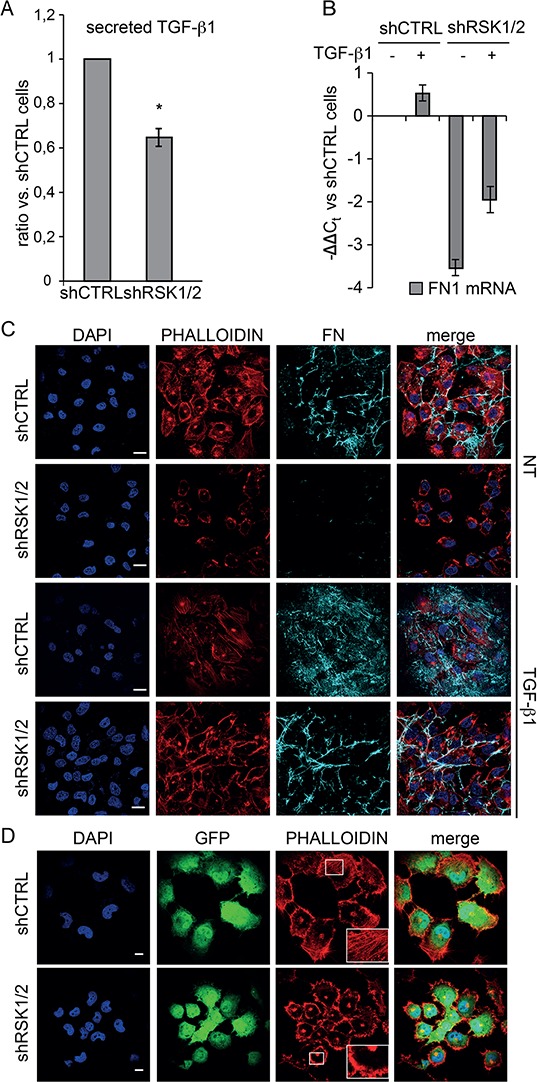
RSK1 and RSK2 silencing affects TGF-β1 secretion, TGF-β1 regulated FN expression and actin organization **A.** measurement of TGF-β1 secretion using MSD technology; **B.** Quantitative Real-time PCR of the expression of FN in cells silenced and treated with TGF-β1; **C.** immunofluorescence confocal analysis of phalloidin, used to visualize actin organization, and FN deposition in control and RSK1/RSK2 silenced cells untreated (NT) or treated with TGF-β1; DAPI was used to visualize cell nuclei; FN was labelled with the IST9 antibody specific for cell derived FN; **D.** immunofluorescence confocal analysis performed as above to study F-actin organization in GFP labelled control and RSK1/RSK2 silenced cells; in the insets details are magnified. Scale bar: 20 μm.

TGF-β1 has long been known as an effective positive transcriptional regulator of FN expression in fibroblasts and other cell types [28, 29] as well as in mesothelial cells during the process of adhesion of ovarian cancer cells to the peritoneum [23]. Of note, we observed how in ovarian cancer cells the secretion of TGF-β1 protein (Figure 7A), but not the transcription of the *TGFB1* gene (not shown), was reduced by RSK1/RSK2 silencing. In addition, the stimulation with exogenous TGF-β1 promoted *FN1* gene transcription and effectively rescued the lack of production of endogenous FN protein in RSK1/RSK2 silenced ovarian cancer cells (Figure 7B–7C). As expected [26, 27], TGF-β1 upregulated FN mRNA and protein in control ovarian cancer cells as well (Figure 7B–7C).

In accordance with the notion that FN is involved in mechanotransduction and actin cytoskeleton organization [30], we found that ovarian cancer cell morphology and polymerized actin organization were profoundly altered by RSK1 and RSK2 knock down (Figure 7D). Compared to control cells, RSK1/RSK2 silenced cells were more rounded, displayed a robust increase in the amount of cortical F-actin and decrease in the amount of stress fibers and filopodia. The rescue of the expression of either RSK1 or RSK2 showed that each kinase alone was able to revert these phenotypic alterations (Supplementary Figure S7A). Moreover, plating silenced cells on exogenous FN fully rescued phenotypic alterations in F-actin polymerization (Supplementary Figure S7B) and cell size (Supplementary Figure S7C and S7D). Accordingly, the defective wound healing of RSK1/RSK2 silenced cells was fixed when cells were plated onto exogenous FN (Supplementary Figure S7E).

### YB-1 activation by p90RSK is necessary to sustain the fibronectin/TGF-β1 dependent pro adhesive circuit in ovarian cancer cells

**Figure 8:**
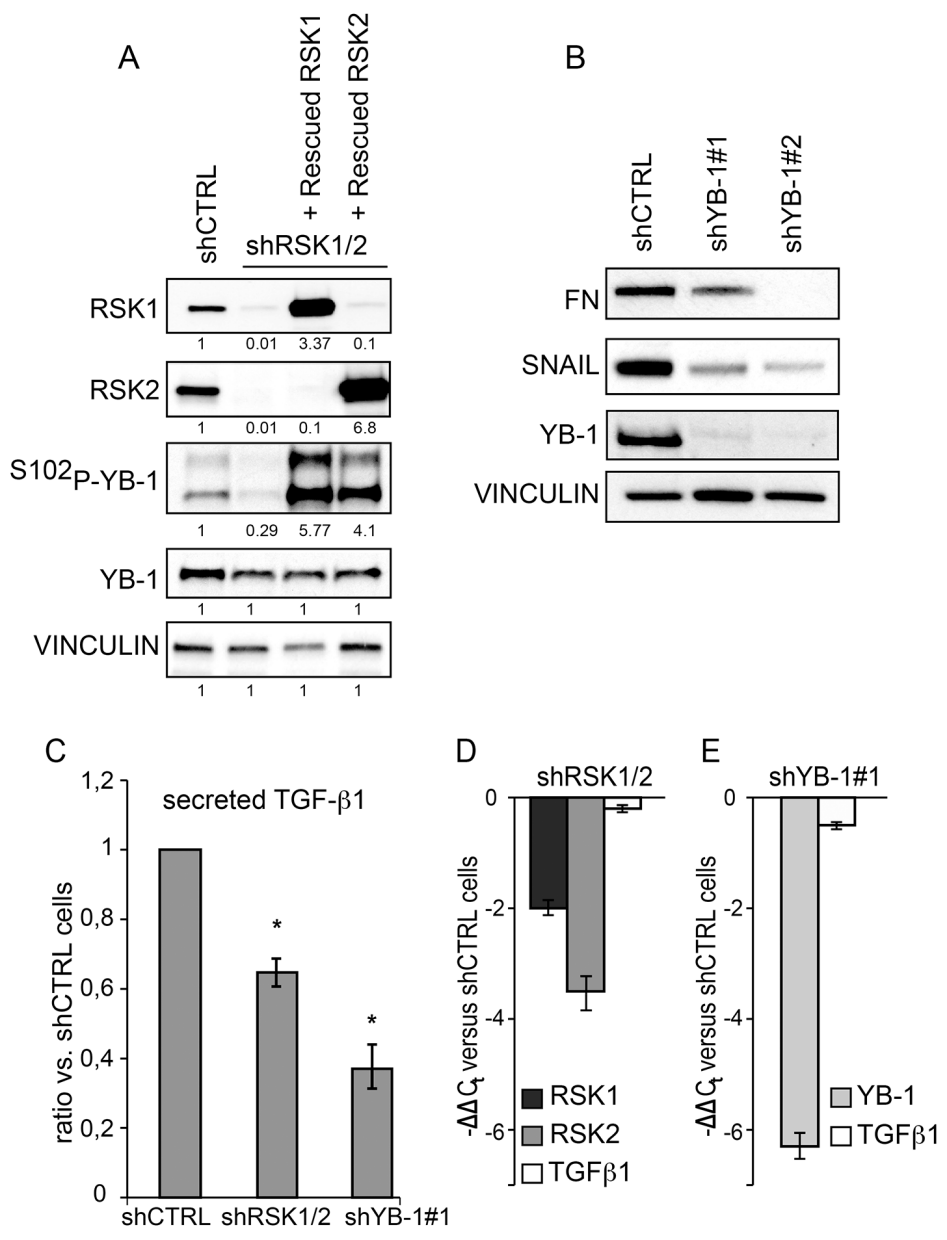
RSK1 and RSK2 silencing affects FN expression and TGF-β1 secretion by impairing YB-1 phosphorylation **A.** Western blot analysis of YB-1 phosphorylation in OVCAR-8 cells silenced and rescued as described in the Legend to Figure 1; RSK1/2 band intensity was quantified versus vinculin; P-YB-1 versus YB-1 **B.** Western blot analysis of fibronectin (FN) and SNAIL in cells where YB-1 expression was suppressed with two different shRNAs; **C.** measurement of TGF-β1 secretion using MSD technology in cells silenced as in the panel (A) and (B); **D–E.** Quantitative Real-time PCR to evaluate the expression at mRNA level of the listed genes in cells silenced as indicated. All blots were re-probed with vinculin antibody to confirm equal loading.

During epithelial to mesenchymal transition (EMT) FN expression is up-regulated by YB-1 [31], which promotes EMT and the expression of EMT markers [31]. In ovary and breast cancer cells YB-1 is phosphorylated, i.e. regulated, by p90RSK [15, 32, 33]. As YB-1 is also targeted by other kinases, we confirmed that in these cells YB-1 phosphorylation (Figure 8A) was: (i) present when RSK1 and RSK2 were both active; (ii) almost abrogated by RSK1/RSK2 silencing and (iii) strongly activated upon rescue of either RSK1 or RSK2 in doubly silenced cells. In line, the expression of the EMT markers ZEB and SNAIL was reduced in RSK1/RSK2 silenced cells, either in basal condition or after the induction of EMT by HGF (Supplementary Figure S8).

The dependence of FN expression on YB-1 in ovarian cancer cells was confirmed by the reduced expression of FN protein upon YB-1 silencing (Figure 8B). SNAIL, the best known target of YB-1, was regulated accordingly (Figure 8B). Moreover, in keeping with the knowledge that YB-1 regulates the translation of TGF-β1 [34], we discovered that YB-1 silencing reduced TGF-β1 secretion (Figure 8C) without affecting its transcription (Figure 8D–8E).

## DISCUSSION

This study demonstrates that RSK1 and RSK2 are critical for ovarian cancer cell ability to establish experimental metastases to the peritoneum and to the lung, likely by regulating the secretion of endogenous FN and FN-dependent activation of α5β1 integrin, and thus cell adhesion that is necessary for invasiveness. Our data suggest that RSK1 and RSK2 might be important in the establishment of metastases of human ovarian cancers in both the peritoneum and in distant organs.

Here, we show that in ovarian cancer cells RSK1 and RSK2 share most functional properties and behave redundantly in most *in vitro* assays. Although a certain level of functional redundancy between the four RSK isoforms was expected, evidence exist for isoform-specificity in mediating cellular processes [for a review see 12] and could be due to expression pattern differences. Here we provide evidence that RSK3 and RSK4, to which tumor suppressive functions have been attributed, were barely detectable in a number of ovarian cancer cell lines, while RSK1 and RSK2 were expressed in all examined cell lines. Isoform specificity might be also ascribed to different downstream signals. *In vitro* evidence suggested that RSK1 is a negative regulator of cell invasion, while RSK2 is pro-migratory and thus pro-metastatic [8]. Several other reports [summarized in ref. 12] are instead in line with our data showing that RSK1 and RSK2 are both active and may be redundant in stimulating motility and invasiveness of ovarian cancer cells.

Our *in vivo* finding showed that RSK1 and RSK2 have a role in both the formation of ovarian cancer peritoneal colonies and metastases to distant organs. In this framework, it is conceivable that the role played by RSK1 and RSK2 in anchorage-independent growth, adhesion, and invasion is crucial for the establishment of metastases. Anchorage independent growth, in particular, appears to be central for metastatic dissemination through the bloodstream [35]. Moreover, 24 hours after the i.v. injection only RSK1/RSK2 proficient cells remained in the lung tissue, a process that most likely requires adhesion during the initial phases of tissue invasion by ovarian cancer cells. The impaired formation of lung metastases after subcutaneous injection of RSK1/RSK2 silenced cells confirmed that this initial homing was obligatory for metastasis. Also after i.p. injection RSK1/RSK2 silenced cells did not complete short term adhesion and hence the establishment of metastases to the peritoneum.

In agreement with the hypothesis suggested by functional data, in ovarian cancer cells RSK1 and RSK2 regulated the expression and function of proteins involved in cell adhesion and spreading, such as FN and its major receptor α5β1 integrin. The role of the pathways upstream and downstream these molecules in ovarian cancer had been shown in elegant *in vitro* models of peritoneal dissemination, such as 3D organotypic culture mimicking the key components of the peritoneal/omental surface microenvironment [36] and ovarian cancer spheroids able to clear the mesothelium [37]. In accordance, we found that F-actin remodeling after adhesion was also affected in RSK1/RSK2 silenced cells, likely because of altered FN secretion and α5β1 integrin-dependent cell adhesion. The involvement of matrix deposition in the *in vivo* homing/adhesion of ovarian cancer cells in the peritoneum has been elegantly demonstrated by other Authors using either floxed *fn1* mice [23] or a function-blocking α5β1-integrin antibody [19].

**Figure 9:**
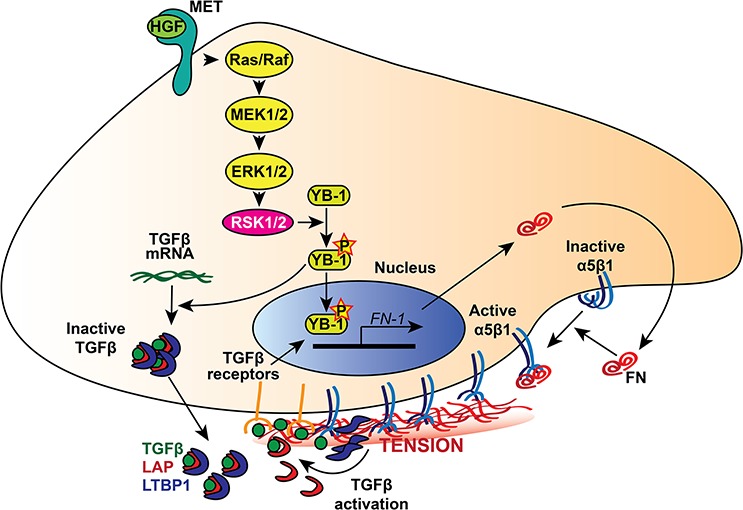
RSK dependent molecular mechanism of FN secretion and integrin activation in ovarian cancer cells The p90RSK encoded by RSK1 or RSK2 is activated downstream the RAS-ERK/MAPK pathway, which could be activated by growth factor, such as HGF that stimulates the Met tyrosine kinase receptor. Activated RSK1/RSK2 kinases drive the phosphorylation of the transcription factor YB-1, which then promotes TGFβ mRNA translation and *FN-1* gene transcription. FN secreted by ovarian cancer cells increases α5β1 integrin activation and enhances tumour cell adhesion, motility, and invasion. Furthermore, ligand-bound integrins stretch FN fibrils and promote the mechanical release of TGFβ from the complex composed of LTBP1 and LAP, which maintains the growth factor in a FN-bound latent form. In turn, activated TGFβ promotes *FN-1* gene transcription, giving rise to a positive feedback loop.

Here, we provide evidence supporting the notion that the mechanism by which ovarian cancer cells achieve homing in a foreign environment and form metastases may be cell autonomous. Indeed, we observed that ovarian cancer cells are *per se* able to secrete endogenous TGF-β1 and FN, the latter being long known to be up-regulated by TGF-β1 itself [28, 29]. It was also already known that FN expression by mesothelial cells is induced by TGF-β1 produced by ovarian cancer cells, via the TGF-β1 receptor/RAC/SMAD dependent transcriptional pathway, and is critical for the metastasis of ovarian cancer cells to the peritoneum [23]. Notably, FN was previously reported to be required for integrin-mediated activation of latent TGF-β1 [38–40], thus leading to a feed-forward loop able to further stimulate FN secretion and TGF-β1 activation. This may logarithmically increase the ability of ovarian cancer cell to adhere, invade and metastasize, as FN has a well-established role in cancer metastasis by promoting mitogenic signaling, adhesion, invasion and angiogenesis [for a review see 41] and is overexpressed in ovarian cancer metastases [23]. The drawing of Figure 9 summarizes the molecular mechanisms hypothesized above.

Furthermore, our finding that RSK1 and RSK2 are important for anchorage independent growth of ovarian cancer cells is likely related to the fact that TGF-β1 may support anchorage independent growth *via* increased FN synthesis and to the role played by endogenous FN in this process [42, 43].

As also previously reported [31], we show that FN expression and relevant function may be regulated by the transcription/translation factor YB-1 that in turn is activated by the p90RSK. We show here that also TGF-β1 translation and secretion depended on the p90RSK. This could be due to YB-1 regulation of translation, as previously demonstrated [34]. Other Authors' findings also suggest that YB-1 might play an important role in mediating RSK driving metastases. Indeed, it has been shown that YB-1 knock down in sarcoma and breast cell lines blocks cell metastatic ability *in vivo* [31, 44].

Remarkably, previous evidences point to YB-1 as an excellent molecular marker of ovarian cancer progression, as it has been found increased in experimental metastases in a murine model and a strong correlation was reported between high P-YB-1 levels in ovarian cancers and the poor outcome of patients [15, 45].

Previous reports [5, 7, 8] and our data suggest a broad functional dependence of cell metastatic phenotype on p90RSK activation, making RSK proteins attractive targets for cancer therapy. Although RSKs are not hotspot for mutation and amplification in cancer, p90RSK is one of the main targets of the ERK1/2 pathway that is oncogenically activated in more than 30% of human cancers. In our experiments, the simultaneous knockdown of both RSK1 and RSK2 was required to phenocopy most of the effects of pharmacological inhibitors. While the extent of functional redundancy of RSK isoforms is not completely understood, it is worth noting that all of them are suppressed by most known inhibitors, FMS being a notable exception [46]. Currently, there are no clinically available RSK inhibitors, yet a few small molecules have been identified through screening efforts [see e.g. 47]. Importantly, RSK inactivation does not appear to be toxic in most cell types, as demonstrated by the viability of *Rsk1*/*Rsk2*/*Rsk3* triple-knockout mice [48]. Should systemic suppression of RSK activity indeed be well tolerated, RSK family members are among the most promising therapeutic targets for the suppression of cancer metastasis.

## MATERIALS AND METHODS

### Cell lines and reagents

HeLa, SK-OV-3, TOV21-G, OVCAR-3, OVCAR-4, OVCAR-5, IGROV-1, OAW42 were purchased from the American Type Culture Collection in 2011. OVCAR-8 cell line was from the NCI-60 collection and obtained from Charles River in 2011. All cell lines have been characterized by the provider and maintained as suggested. Autocrine HGF loop was established in OVCAR-8 cells by transducing cells with a lentiviral vector carrying HGF transgene [49]. Human recombinant SF1/HGF and TGF-β1 were purchased from R&D Systems (Minneapolis, MN, USA).

Stable silencing of *RSK1* and *RSK2* was achieved using the RSK1 and RSK2-specific human TRIPZ lentiviral inducible shRNA and pGIPZ lentiviral shRNA (Clone V2LHS_241402, V2LHS_47382, V2THS_241402, V2THS_47382, respectively, Open Biosystems, Huntsville, AL, USA). Stable silencing of *YBX1* was achieved using the YBX1-specific human MISSION^®^ shRNA (TRCN0000315307, TRCN0000315309). As a control, cells were transduced with lentiviral particles carrying a non-targeting short hairpin sequence (shCTRL). Stable expression of luciferase was obtained using the pRRL.sin.PPT.CMV.Luciferase.pre vector.

For RSK1 and RSK2 rescue, RNA interference resistant *RSK1* and *RSK2* cDNA were obtained by the insertion of 8 different silent mutations specific for the shRNA used, into the RSK1 and RSK2 cDNA sequence. All the mutations were introduced using a PCR- based technique as described elsewhere [50]. Briefly, the RSK1 and RSK2 mutant cassettes were substituted in the RSK1 and RSK2 cDNAs cloned into pDONR-223 Gateway^®^ Entry vector from Human Kinase ORF collection (Addgene). Then, RSK1 and RSK2 mutant cDNAs were subcloned into the pCCL.sin.PPT. hPGK.GFP.Wpre lentiviral vector.

### Cell transduction with lentiviral vectors

Cells were transduced using second generation lentiviral vectors as described elsewhere [51].

### Western blot analysis

Western blot analysis was carried out as described elsewhere [49]. The following antibodies were used: rabbit polyclonal anti-β-tubulin (H-235) and mouse monoclonal anti-fibronectin (IST-9) from Santa Cruz Biotechnology (Santa Cruz, CA, USA); rabbit polyclonal anti-Integrin α5 (AB1949) and goat polyclonal anti-HGF from R&D System (Minneapolis, MN, USA); rabbit polyclonal anti-RSK1, rabbit monoclonal anti-RSK2 (D21B2) XP, rabbit monoclonal anti-phospho-p90RSK (380), rabbit monoclonal anti-phospho-RSK2 (227) (D5EA11), rabbit polyclonal anti-YB1 (D299), rabbit monoclonal anti-phospho-YB1 (Ser102) (C34A2) and rabbit monoclonal anti-SNAIL (C15D3) obtained from Cell Signaling Technology (Beverly, MA, USA); mouse monoclonal anti-Vinculin (hVIN-1) from Sigma, Saint Louis, MO, USA. It is worth noting that two bands of YB-1 become visible when the level of YB-1 expression is very high as in the case of rescued cells. Indeed, two bands are recognized by several antibodies and are differently interpreted [see e.g. ref. 52].

Bound antibodies were detected using the appropriate peroxidase-conjugated secondary antibody and revealed by enhanced chemiluminescence (Pierce, Rockford, IL, USA).

### Quantitative RT-PCR

Quantitative PCR was carried out as described elsewhere [51]. Briefly, total cellular RNA was isolated using the SV Total RNA Isolation kit (Promega, Fitchburg, WI, USA). To quantify the expression levels of the genes of interest, equal amounts of cDNA were synthesized using the Moloney murine leukemia reverse transcriptase (Promega) and mixed with SsoFast EvaGreen Supermix (Bio-Rad, Hercules, CA, USA) and 300 μM of each of the respective forward and reverse primers. Quantitative real-time PCR was done on a MyiQ thermal cycler (Bio-Rad). Each target gene expression was evaluated using a relative quantification approach, with PPIA (GenBank accession no. NM_021130.4) as an internal reference. Primer sets used are as follows: PPIA: forward CATCCTAAAGCATACGGGT, reverse TTCTTGCTGGTCTTGCCATT; RPS6KA1: forward GGAGGGCCACATCAAACTCA, reverse GGACCACCAGTCCGCACTAT; RPS6KA2: forward GAAGGCTGCGACATCTGGAG, reverse TGTTCCAATTTCCCCCACTG; RPS6KA3: forward, GAAGGCCACACTGAAAGTTCG reverse TCCTCCCCTGAGAAAATCCAA; RPS6KA6: forward GCCGAGGCGGTGGATC, reverse ACAGGAGAATCACTTGAACCTGG. Human FN1 primers for quantitative RT-PCR were purchased from Bio-Rad (Hercules, CA, USA). PCR cycling conditions were as follows: 30 s at 95°C 30, 5 s at 95°C plus 15 s at 60°C (40 cycles), 30 s at 95°C, and 10 s at 65°C plus 10 s at 0.5°C (60 cycles: melting curve).

### Viability assay

*In vitro* growth curve was determined using the CellTiter Glo proliferation assay (Promega, Madison, WI, USA) according to the manufacturer's protocol.

### Soft agar assay

SK-OV-3 (6000 cells/well) and OVCAR-8 (2000 cells/well) cells were seeded in triplicate in 6-well plates in a 0.5% SeaPlaque agar layer (Lonza, Walkersville, MD). The plates were incubated for 4 and 2 weeks for SK-OV-3 and OVCAR-8 cell lines, respectively. Colonies were stained with MTT salts (Sigma-Aldrich, Saint Louis, MO), photographed, and counted using NIH ImageJ (W. Rasband, NIH) software. Three independent experiments were performed.

### Functional *in vitro* assays

Wound healing, motility and Matrigel^®^ invasion assays, invasion of three-dimensional collagen gel were done as described [53].

### Immunofluorescence

Cells, grown on glass coverslip, were fixed in 4% paraformaldehyde for 20 min at room temperature and permeabilized with 0.1% Triton-X100 in PBS for 2 min on ice. Then cells were treated at room temperature with 1% BSA in PBS for 30 min and incubated for 1 h at room temperature with the following primary antibodies diluted in PBS containing 1% donkey serum for 1 h.: mouse anti-fibronectin IST-9 (Santa Cruz, CA, USA) or mouse monoclonal anti-integrin alpha5 (Preservative Free) (SNAKA51) (Millipore Darmstadt, Germany) or rabbit monoclonal anti-paxillin (NT) (clone Y113) (Millipore Darmstadt, Germany). After washing, cells were fluorescently labeled, according to the primary antibody used, with an Alexa Fluor^®^ 647, Alexa Fluor^®^ 555 donkey anti-mouse antibody or Alexa Fluor^®^ 555 donkey anti-rabbit antibody (Molecular Probes, Eugene, OR, USA) diluted 1:400 in PBS containing 1% donkey serum for 1 h. Nuclei were stained with DAPI. F-actin was stained with Alexa Fluor^®^ 555 or Alexa Fluor^®^ 647 Phalloidin (50 μg/ml). Coverslips were then mounted using the fluorescence mounting medium (Dako, Glostrup, DK) and analyzed using a confocal laser scanning microscope (TCS SPE II; Leica, Wetzlar, D) equipped with 63 ×/1.40 oil immersion objective.

### Measurement of TGF-β1 secretion

One × 10^6^ control and RSK1/RSK2 stably silenced cells were plated and grown in medium without serum for 48 hours. Supernatants were collected and TGF-β1 was measured using the electrochemiluminescence-based ELISA kit (K151IUC-1) from MSD (Rockville, MD, USA). Protocol and reagents supplied by MSD were used.

### Short term lung colonization assay

Animal studies were carried out following the guidelines of the Italian Ministry of Health. Protocols were approved by the local Veterinary Ethic Committee, the local health authorities and by the Italian Ministry of Health.

Short-term lung colonization assay was carried by labelling 5 × 10^5^ OVCAR-8 control cells with cellTrace™ VIOLET (Molecular probes) and 5 × 10^5^ RSK1/RSK2 silenced OVCAR-8 cells with cellTrace™ CFSE (Molecular probes, USA). Cells were mixed in 200 μl PBS and three mice (NOD/SCID) for each time point were injected intravenously. Lungs were explanted 2, 24 and 48 hours after injection and fixed. Microscopy imaging was performed using a Leica TCS SP5-AOBS 5-channel confocal and multiphoton system (Leica Microsystems) equipped with a pulsed femtosecond Ti:Sapphire (Ti-Sa) Chameleon Vision II laser (Coherent), tunable for excitation from 680 to 1080 nm. Violet, CFSE and dsRED were captured by two detection channels. The two-photon laser was tuned to a wavelength of 700 nm and 985 nm for the first and second channel respectively. Emission wavelengths of 400–476 nm (blue, Violet), 490–539 nm (green, CFSE), and 589–718 nm (red, for dsRED) were collected. Images of the tissues were taken using a HCX PL APO CS 20 ×/0.7 NA oil immersion objective. Series of x-y-z images (typically 0.75*0.75*2.5 μm^3^ voxel size) were collected along the z-axis at 2.5 μm intervals throughout the tissues (30–50 μm) depth images were then analyzed with ImageJ software. The experiment was repeated three times.

### Haematogenous spontaneous metastasis

Two × 10^6^ OVCAR-8 cells, engineered to secrete HGF and then transduced with an inducible lentiviral vectors (pTRIPZ) to express either shRNA for RSK1 and RSK2 or control shRNA (shCTRL), (characterized by the TurboRFP that marks cells expressing shRNA), were injected subcutaneously (s.c.) in the right posterior flank of 6-week old NOD/SCID mice. After 4 weeks the expression of vector was induced by adding doxycycline in the drinking water of mice. Tumours were measured using a caliper each week and volume calculated using the formula (Dxd^2^)/2, where D is the major tumour axis and d is the minor tumour axis.

After 8 weeks animals were sacrificed. Lungs were insufflated with India ink dye, removed, washed and bleached in Fekete's solution before counting colonies under a stereo microscope.

### *In vivo* adhesion assay

Four × 10^6^ OVCAR-8 control or doubly silenced cells (transduced using shRNA driven by lentiviral vectors carrying GFP and also engineered to express luciferase enzyme) were injected intra-peritoneum in NOD-SCID mice. After 4, 48 and 120 hours mice were sacrificed and peritoneum, omentum, diaphragm and abdominal fat were excised, washed in PBS and then lysed with NP-40 1%. The number of attached cells was quantified by measuring the fluorescent intensity with a fluorescence spectrophotometer (Synergy HT) and using as negative control tissues of mice injected with non fluorescent cells.

### Peritoneal metastasis assay

Four × 10^6^ OVCAR-8 control or doubly silenced cells (transduced as for the *in vivo* adhesion assay) were injected intra-peritoneum in NOD-SCID mice. Luciferin i.p. injection (150 mg/Kg body weight) and bioluminescence were used to monitor peritoneal metastasis formation through time by means of the IVIS^®^ Lumina II imaging system (Caliper Life Sciences, Hopkinton, MA, USA). After 21 days mice were sacrificed and formation of metastatic nodules was evaluated by measuring fluorescence intensity with the IVIS instrument. The area covered by metastasis was quantified using ImageJ software.

### Statistical analysis

Statistical analysis of the data was performed using ANOVA (Microsoft Excel; Microsoft, Redmond, WA, USA).

## SUPPLEMENTARY FIGURES


