# Supplementary material for: Peritoneal and hematogenous metastases of ovarian cancer cells are both controlled by the p90RSK through a self-reinforcing cell autonomous mechanism
Source: Oncotarget. 2015 Nov 26;7(1):712–28. doi: 10.18632/oncotarget.6412 (PMC4808028; doi:10.18632/oncotarget.6412)
Supplement: Supplementary file 1 [file oncotarget-07-0712-s001.pdf]

SUPPLEMENTARY FIGURES

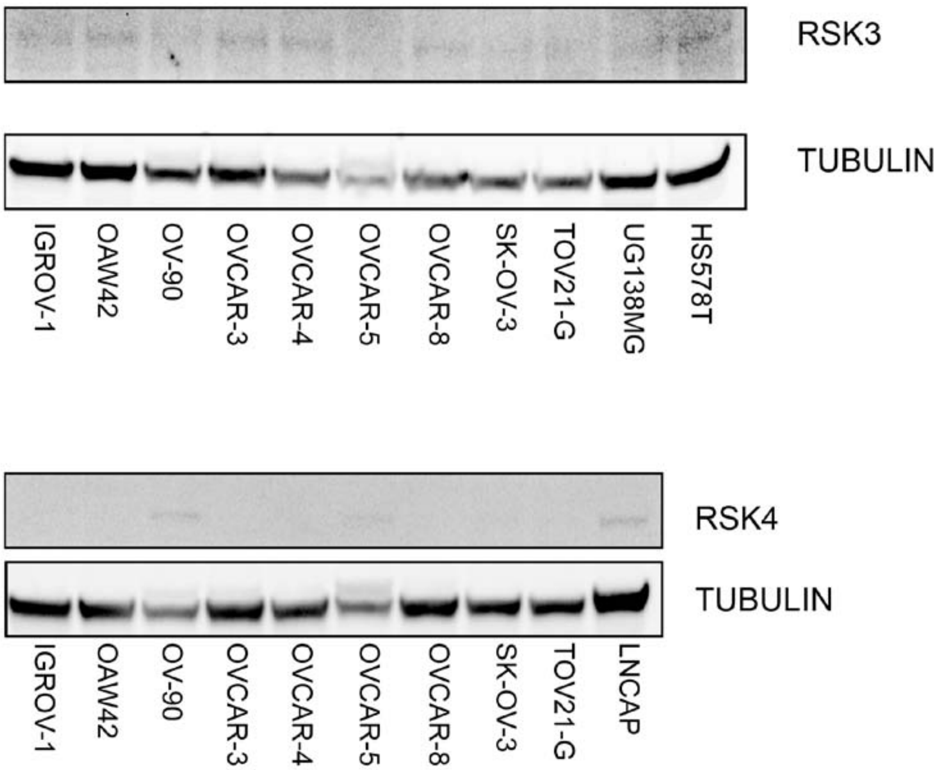

Supplementary Figure S1: Expression of the RSK3 and RSK4 in the ovarian cancer cell lines where expression of RSK1 and RSK2 has been studied and shown in Figure 1. Western blot analyses showing RSK3 and RSK4 protein expression; blots were reprobed with tubulin antibody to confirm equal loading. The HS578T breast cancer and the glioblastoma UG138MG cell lines have been analyzed as positive controls for the expression of RSK3; the prostate carcinoma LNCAP cell line was the positive control for the expression of RSK4. These cell lines are reported as positive controls, although it is known that the two genes are expressed at low level in all cell lines (see the following Supplementary Figure S2).

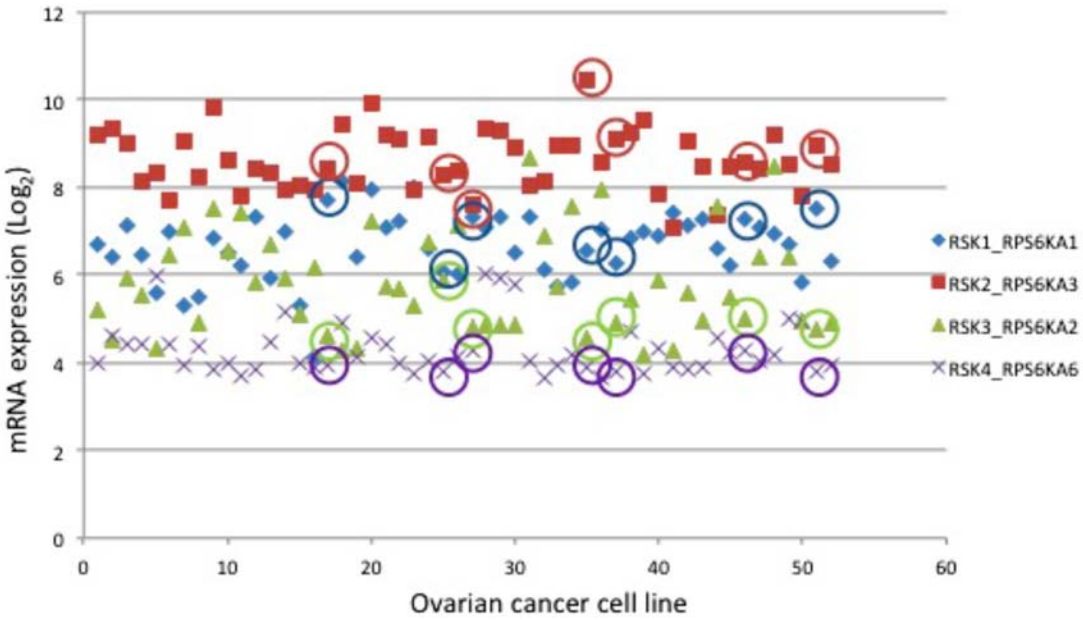

Supplementary Figure S2: Expression of the four genes encoding RSK1/2/3/4 proteins in the 52 ovarian cancer cell lines analysed and reported in the Cancer Cell Line Encyclopaedia. (CCLE. https://www.broadinstitute.org/software/cprg/?q=node/11). The cell lines studied at protein level and shown in Figure 1 and Supplementary Figure S1 are surrounded by circles.

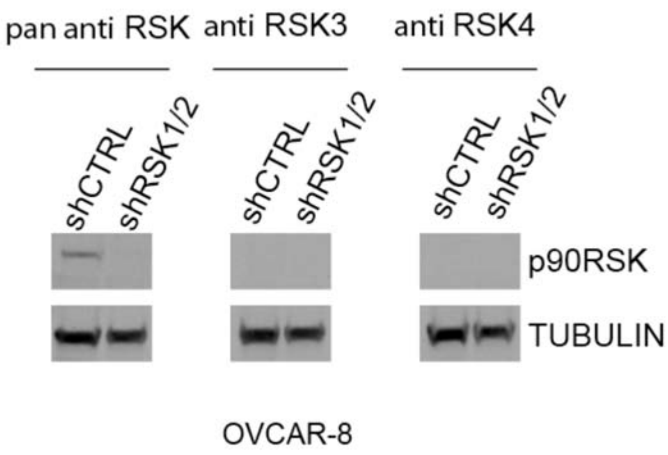

Supplementary Figure S3: Control of expression of RSK3 and RSK4 protein products in OVCAR-8 cells where RSK1 and RSK2 have been silenced. Western blot analyses of silenced cells using a pan-RSK antibody and RSK3 and RSK4 specific antibodies. Blots were reprobed with tubulin antibody to confirm equal loading.

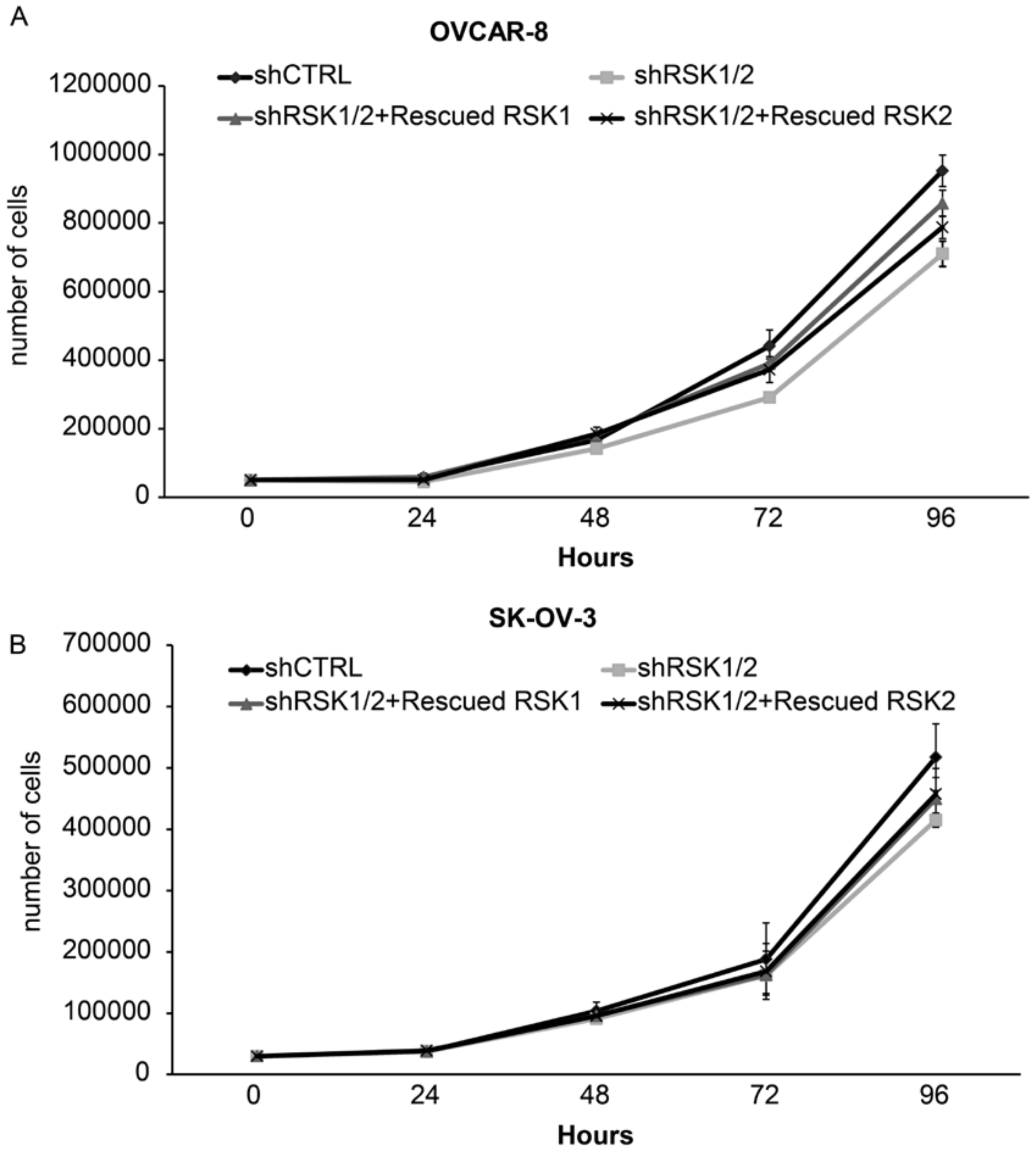

Supplementary Figure S4: Growth curves of RSK1 and RSK2 silenced ovarian cancer cells. OVCAR-8 **A.** and SK-OV-3 **B.** cells were transduced to express simultaneously the RSK1 and the RSK2 specific shRNAs (shRSK1/RSK2) or control scramble shRNA (shCTRL) and subsequently to express either RSK1 or RSK2 sh-resistant cDNA. Cells were plated in complete FBS containing medium and counted every 24 hours.

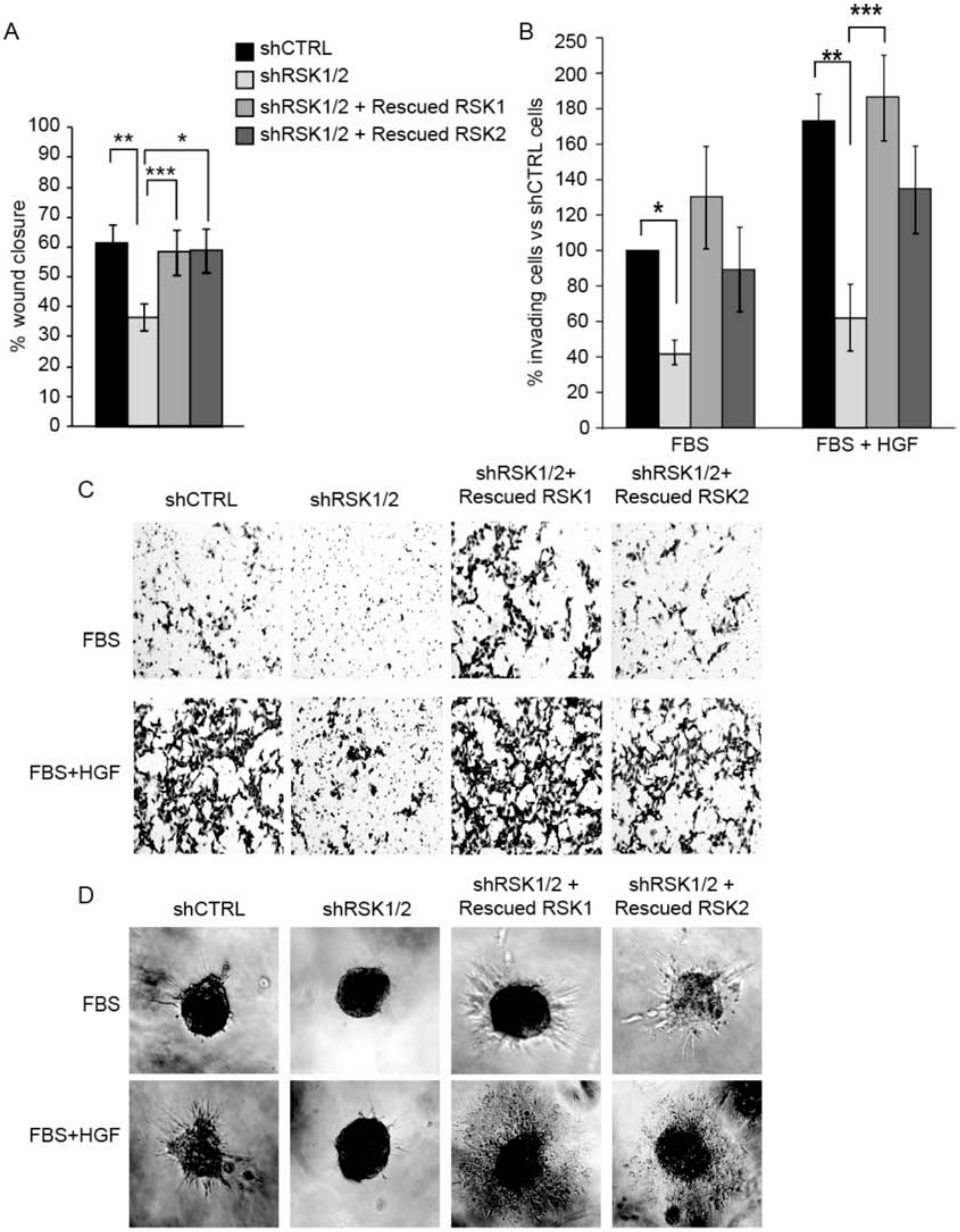

Supplementary Figure S5: Rescue of either RSK1 or RSK2 in RSK1/RSK2 silenced OVCAR-8 cells to evaluate the specificity of functional effects of silencing and the contribution of either kinase to cell motility and invasiveness. A. Wound healing assay to evaluate the migration towards the acellular area of cells engineered as shown in Figure 1 and exposed to 50 ng/ml HGF for 24 hours. **B–C.** Matrigel® invasion assay: representative images (C) and quantification (B) of cells invading in 16 hours the lower chambers of Transwells covered with Matrigel®, in the presence of either foetal bovine serum (FBS) or FBS plus HGF, taken with optical microscopy after cell staining with crystal violet. **D.** 3D collagen assay: representative images of cells engineered as above and embedded in 3D collagen matrix as in the Legend to Figure 2, taken with phase contrast microscopy. Statistical significance was determined using ANOVA test: *$*P < 0.05$, $**P < 0.01$, $***P < 0.001$.*

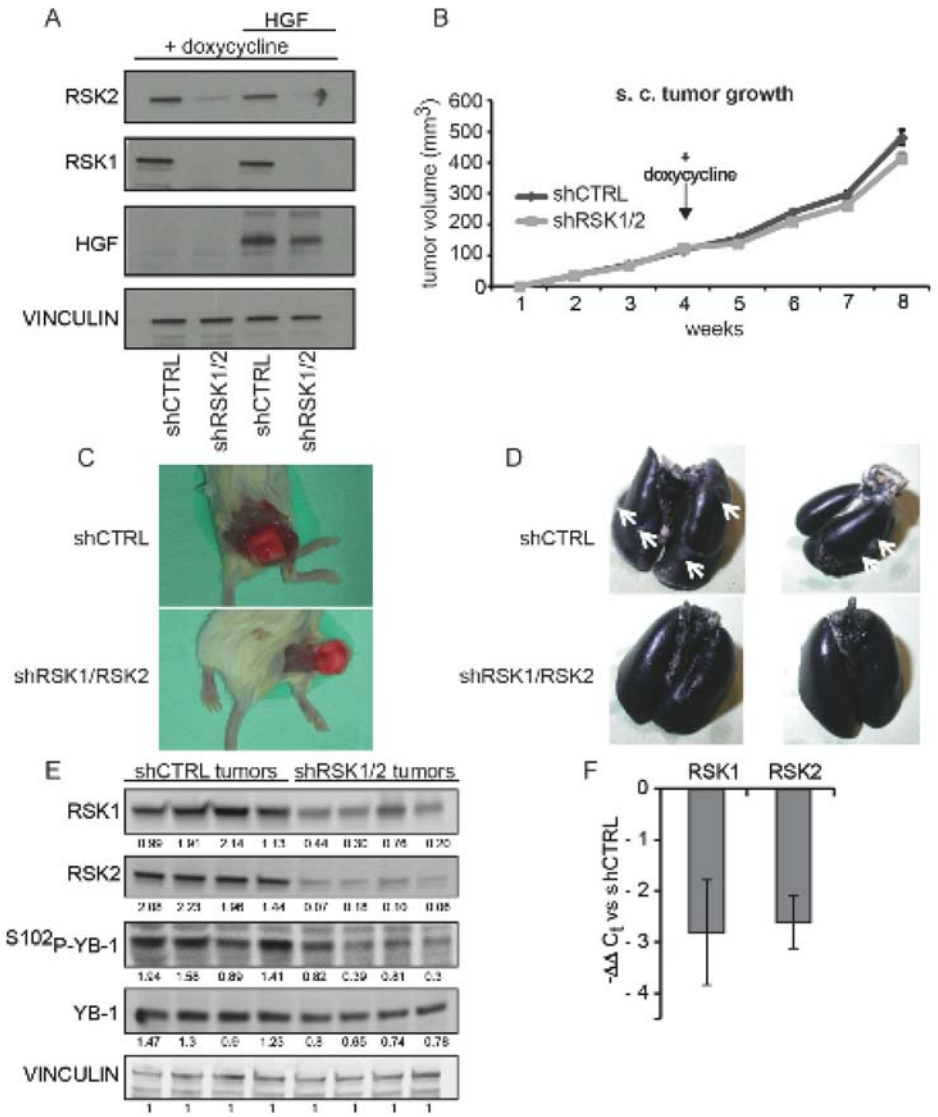

Supplementary Figure S6: Growth as xenografts and metastasis of the OVCAR-8 cells engineered to express inducible RSK1/RSK2 or control shRNAs and HGF. A. Western blot analysis to show the induction of RSK1 and RSK2 specific shRNAs, control shRNA and the expression of HGF in control and silenced cells. **B.** Two × 10^6 OVCAR-8 cells engineered as above were injected subcutaneously in the right posterior flank of 6-week old NOD/SCID mice. After 4 weeks, when tumour volume was approximately 100 mm^3, RSK1 and RSK2 silencing was induced by adding doxycycline in the drinking water of mice. After 8 weeks mice were sacrificed. **C–D.** Muscle invasion (C) and lung metastascs (D) in a representative mouse with xenografts, **E–F.** Expression of RSK1 and RSK2, at either protein (E) or mRNA (F) level in the relevant xenografts.

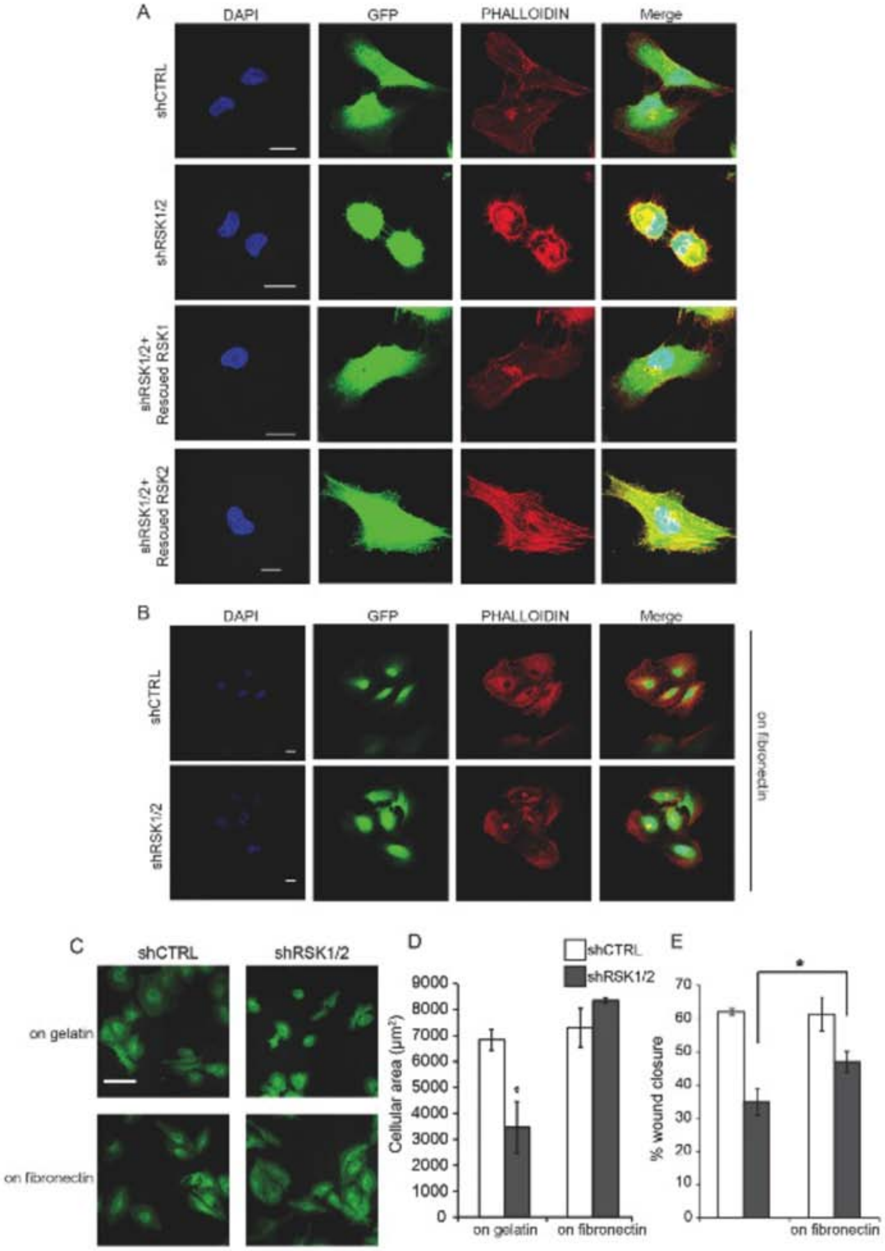

Supplementary Figure S7: Rescue of the phenotypic alterations caused by RSK1/RSK2 silencing after re-expression of either RSK1 or RSK2 or cell plating cells onto exogenous fibronectin (FN). A. and **B.** immunofluorescence confocal analysis of the organization of F-actin visualized with phalloidin in GFP labelled control and RSK1/RSK2 silenced OVCAR-8 cells. Scale bar: 20 μm; **C.** Control or RSK1/RSK2 silenced cells were seeded on gelatin or on 10 μg/ml fibronectin. After 22 hours cells were fixed with glutaraldehyde and stained with crystal violet. The presence of crystal violet dye was captured by means of widefield fluorescence microscopy (excitation: 570/20; emission 640/40) in different fields. Images were processed with background subtraction function of ImageJ software. Monochromatic images are shown in table (Scale bar 100 μm), while in **D.** average cellular area was calculated by dividing total fluorescent cellular area by total cell number in each field. Error bars represent the standard deviation of cellular area among different fields; **E.** wound healing assay in the presence of foetal bovine serum (FBS) of cells plated either onto plastic or onto exogenous fibronectin; 24 hours after wounding, cells that migrated to the acellular area were photographed under the microscope and percentage of closure was measured with ImageJ software.

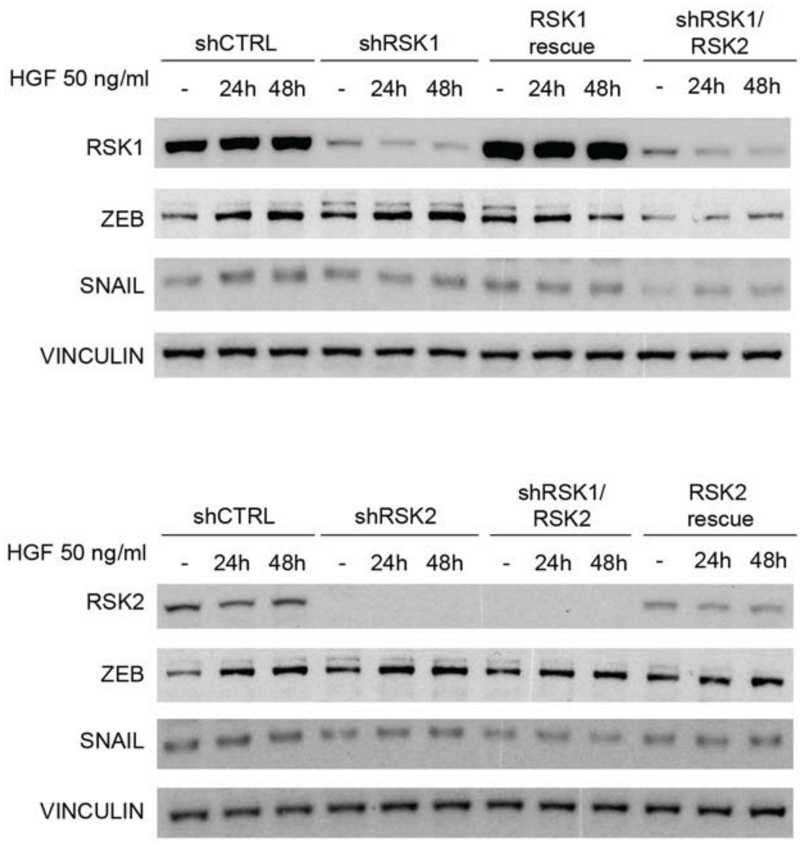

Supplementary Figure S8: Expression of the EMT markers ZEB and SNAIL in OVCAR-8 cells where either RSK1 or RSK2 or both proteins were silenced. Western blot analysis was carried out of cells grown in basal condition or after cell exposure for the indicated times to HGF, which is known to induce scatter effect, i.e. phenotypic epithelial to mesenchymal transition of epithelial cells.
